# Enteroviruses and Type 1 Diabetes Mellitus: An Overlooked Relationship in Some Regions

**DOI:** 10.3390/microorganisms8101458

**Published:** 2020-09-23

**Authors:** Abdulaziz Alhazmi, Famara Sane, Mouna Lazrek, Magloire Pandoua Nekoua, Francis Badia-Boungou, Ilka Engelmann, Enagnon Kazali Alidjinou, Didier Hober

**Affiliations:** 1 Laboratoire de Virologie ULR3610, Univ Lille, CHU Lille, F-59000 Lille, France; abalhazmi@jazanu.edu.sa (A.A.); famara.sane@chru-lille.fr (F.S.); mouna.lazrek@chru-lille.fr (M.L.); pamanek88@gmail.com (M.P.N.); badiaboungounzatsifrancis@yahoo.fr (F.B.-B.); ilka.engelmann@chru-lille.fr (I.E.); enagnonkazali.alidjinou@chru-lille.fr (E.K.A.); 2 Microbiology and Parasitology Department, College of Medicine, Jazan University, Jazan 45142, Saudi Arabia; 3 Laboratoire de Biologie et Physiologie Cellulaires, Institut des Sciences Biomédicales Appliquées (ISBA), Faculté des Sciences et Techniques (FAST), Université d’Abomey-Calavi, 01 BP 526 Cotonou, Benin

**Keywords:** enterovirus, type 1 diabetes mellitus, Middle East, North Africa, Asia, Cuba

## Abstract

Enteroviruses (EVs) infect millions of people annually. EV infections can be asymptomatic or symptomatic with conditions ranging from mild illnesses to serious diseases such as dilated cardiomyopathy. A causal relationship between EV infections and type 1 diabetes mellitus (T1DM) has been heavily debated, with some studies suggesting that this relationship is not yet conclusive and requires additional evidence, whereas others strongly argue for this correlation. While this relationship is well investigated in some developed countries like the USA and Finland, it is understudied or neglected in other countries like Russia for many reasons such as the low incidence of T1DM. Although the Middle East and North Africa (MENA) are highly affected by T1DM, the role of EVs in the disease in MENA has not been investigated extensively. Therefore, we aimed to address the relationship between T1DM and EVs in MENA and other regions globally.

## 1. Introduction

Enteroviruses (EVs) are a group of small, single-stranded, positive-sense RNA viruses belonging to the Picornaviridae family. In general, EVs can infect various tissues of the human body, in any given community and at any time of the year [1]. However, the seasonality of EV infections is notable; they are predominant in summer and autumn in the Western countries and in winter and spring in the Eastern countries [2]. These infections vary greatly in severity and clinical manifestations depending on different factors, such as the infection site and virus serotype. For example, EVs are the most common cause of central nervous system (CNS) infections in children, and most infections are self-limited [3], whereas they can also cause serious cardiac infections, which can be life-threatening [4]. Some chronic diseases have been linked to EVs, such as dilated cardiomyopathy and type 1 diabetes mellitus (T1DM) [5,6,7,8,9].

T1DM is a multifactorial immune-mediated disease characterized by the disruption of insulin-producing cells, i.e., β-cells, in the pancreas. T1DM results from the amalgamation of different genetic and environmental factors. The genetic background of T1DM is complex, as a few genes have found to be causative, whereas others have found to be protective. For example, IDDM1, located on human leukocyte antigen (HLA) II on chromosome 6, is the principal gene linked to most T1DM cases. However, it is difficult to explain the genetic background of T1DM based on the involvement of HLA alone because not all T1DM cases share similar genetic mutations [10]. Moreover, environmental triggers, like dietary agents and viral infections, also play a key role in the pathogenesis of T1DM. EV infections have been extensively linked to T1DM etiology. Studies on human and animal models found that EVs can infect insulin-producing cells [5,11,12,13]. Moreover, EV antibodies and EV RNA are frequently reported in newly diagnosed patients with T1DM [7,14]. Consequently, the relationship between T1DM and EVs has been investigated in many regions highly affected by T1DM.

**Figure 1:**
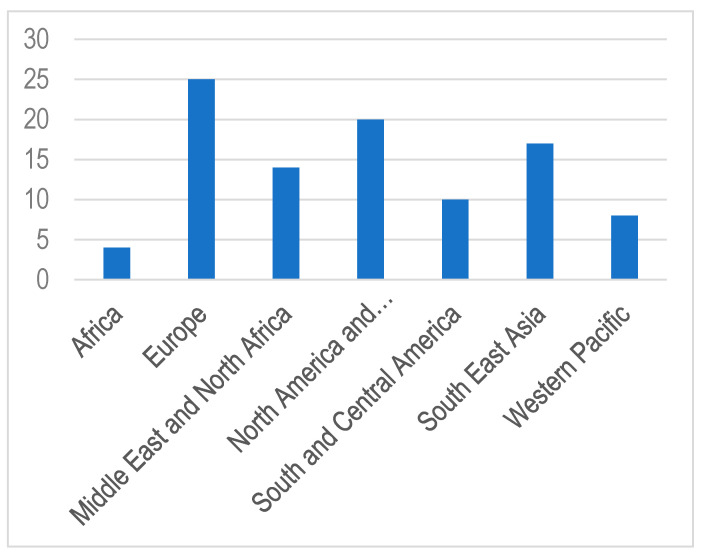
Estimated annual incidence of type 1 diabetes (T1DM) in children and adolescents (0–14 years) in thousands [15].

**Figure 2:**
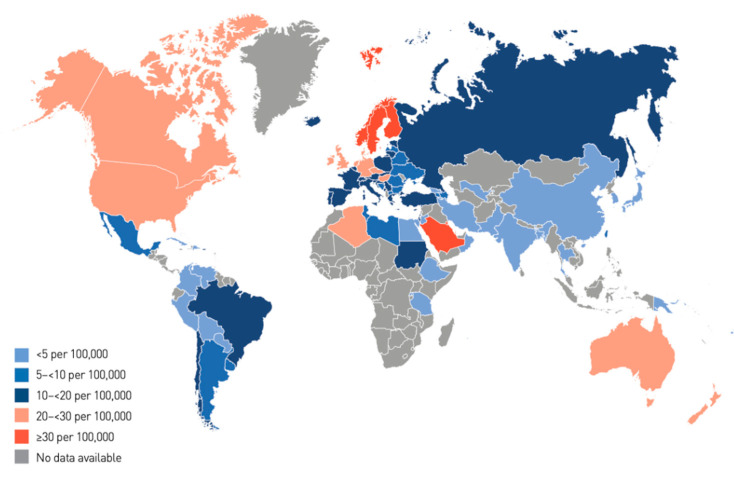
Global incidence of type 1 diabetes in children aged 0–14 years [15].

**Figure 3:**
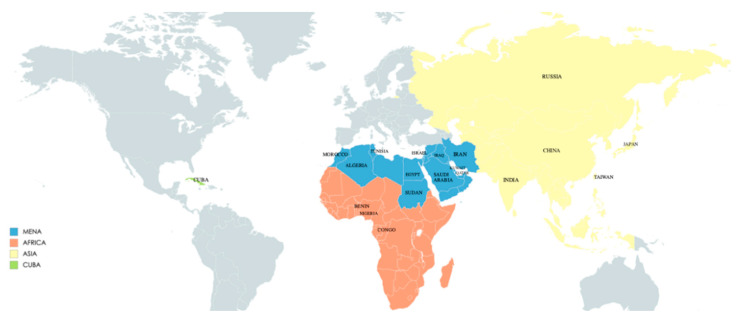
Regions and countries cited in this review.

The annual increase in the number of children and adolescents with T1DM despite the geographic variations is estimated to be 3–4% [15]. In 2019, the global prevalence of T1DM in children and adolescents aged <20 years was 1,000,000 with 129,000 newly diagnosed cases each year. Europe and North America and the Caribbean are the most affected regions, with an incidence rate of 25,000 and approximately 20,000 cases per year, respectively (Figure 1). Regarding the incidence per 100,000 children aged <15 years per year, three countries from Europe (Finland, Sweden, and Norway) and two countries from the Middle East (Kuwait and Saudi Arabia) are the top five countries with an incidence rate of T1DM higher than that of the rest of the countries worldwide. (Table 1; Figure 2 and Figure 3). 

India, Brazil, the USA, and China recorded the highest number in terms of incidence and prevalence of T1DM in children aged <15 years per year [15]. Studies on the causal association between EVs and T1DM revealed that EVs are strongly linked to the development of T1DM, as the time of seroconversion of EV autoantibodies and the development of T1DM share similar seasonal patterns, i.e., in summer and autumn in Europe and the USA [12,16,17,18,19,20]. However, this relationship has not been sought in some other countries, such as Saudi Arabia and Kuwait, despite the high incidence of T1DM. In this review, we included articles that discussed the potential relationship between T1DM and EVs in countries that recorded a high incidence of T1DM. We excluded studies focusing on the USA and European countries as this relationship has already been investigated extensively in those regions.

## 2. MENA

The Middle East and North Africa (MENA) are among the highly affected regions of T1DM incidence. In 2019, 149,400 children and adolescents were diagnosed with T1DM, with 20,800 newly diagnosed cases each year in the MENA region [15]. The rate of incidence in MENA countries is highly variable. For example, in 2019, the incidence rate was 2.5 per 100,000 people in Oman, while it was 41.7 per 100,000 people in Kuwait [15]. Saudi Arabia, which is the largest country in the Middle East, had the highest number of children affected with T1DM, accounting for approximately one-quarter of those in the Middle East, and recorded the highest number of newly diagnosed patients with T1DM at 3700 cases in 2019. Moreover, Kuwait, Saudi Arabia, and Qatar are countries with the highest incidence rate of T1DM in MENA region (Table 1) [21]. However, data on the epidemiology of T1DM in the Middle East are not reliable. For example, in the last five years, Kuwait is the only country that has conducted a nationwide study. Additionally, data in Saudi Arabia and Qatar are partially evaluated based on the results of the oral glucose tolerance test [21]. However, this test is used less because of its disadvantages such as low reproducibility and a time-consuming technique [22,23]. In addition to the high incidence rate, countries in the Middle East share the same seasonality pattern, i.e., predominance in winter and early spring [24,25,26]. 

### 2.1. Kuwait, Saudi Arabia, Qatar and Egypt

A recent study has compared the incidence of T1DM in Kuwait between 2011 and 2013 with that recorded between 1992 and 1997. The incidence has doubled in the last two decades, and further investigation was recommended to identify the cause for this finding [25]. Regarding EV infections in Kuwait, a report has found that EVs are mainly responsible for neonatal sepsis. EVs were detected in the serum of 24% (34 out of 139) of neonates who mainly presented with carditis, sepsis-like illness, or multi-organ failure [27]. Coxsackievirus B was the most isolated serotype. Interestingly, the potential effect of declining maternal neutralizing antibodies against EVs on the high prevalence of neonatal sepsis was discussed. It would be certainly interesting to evaluate the relationship between the low level of maternal neutralizing antibodies against EVs and the high incidence of T1DM in their offspring [27]. 

Different cities in Saudi Arabia have reported that there is a significant increase in the incidence and prevalence of T1DM in the last 20 years [28,29]. A systematic review in 2017 reported that the number of children with T1DM is 16,100. Moreover, this rate of incidence was more likely linked to environmental factors, particularly viral infections, rather than genetic factors. Hence, the authors recommended conducting further epidemiological studies to screen environmental triggers, especially viral infections [29,30]. However, an earlier report in the Eastern region of Saudi Arabia found that there was no relationship between the etiology of T1DM and viral infections, despite the previously noted seasonal pattern of T1DM [31]. In the capital of Saudi Arabia, Riyadh, another early study investigating EV infections during 1989–1995 found that they can occur year-round with a higher incidence in autumn. Echovirus (31 serotypes) and Coxsackievirus B (17 serotypes) were the most common serotypes isolated using cell culture. Moreover, as expected, it was noticed that infants and children were the most susceptible populations [31]. However, whether EV infections in children have a potential impact on the development of T1DM in Saudi Arabia, one of the most affected countries, remains to be elucidated. 

In Qatar, the incidence rate of T1DM is high. It increased from 23.15 (2006–2011) to 28.39 (2012–2016) per 100,000 people, with the reason for this increase not clarified [26]. Another study from Qatar focused on EVs as a common cause of CNS infections and found that they were the most common causative organisms (42%). Notably, most patients were neonates and children (47%), and 5% of them were diagnosed with diabetes. However, not mentioned are the type of diabetes (T1DM or type 2 DM) nor the relationship between T1DM and EVs [32].

Egypt, which is the most populated Middle Eastern country, recorded a lower prevalence of T1DM than other Middle Eastern countries. A study from the Northern region reported that there was a significant increase in the rate of incidence of T1DM per 100,000 people, from 0.7 in 1994 to 3.1 in 2011. Like many countries in the Middle East, T1DM in Egypt was predominant in winter and early spring. However, seasonal viral infections, mainly EVs, could play a role in T1DM etiology [33]. A recent report investigated EVs in 1000 stool samples from children aged <14 years in different regions in Egypt using cell culture, followed by molecular biology techniques for negative cell culture samples. Most EV positive samples (18%) came from individuals residing in rural areas and were reported in spring, thus sharing its seasonality with that of T1DM [34]. However, whether any of those children were diagnosed with T1DM is unknown.

### 2.2. Iraq

Iraq also reported a high incidence of T1DM with approximately 8000 infected children and adolescents in 2019 [15]. According to a recent report, a moderate increase in the incidence and prevalence of T1DM was observed, compared with other countries in the Middle East [35]. A matched case-control study was conducted to investigate the relationship between EVs and T1DM in Iraq (Table 2). The authors used immunoassays to detect IgM and IgG against Coxsackieviruses B3 and B4 and Glutamic acid decarboxylase 65 (GAD65) autoantibodies in serum samples. Additionally, they used RT-PCR to detect Coxsackieviruses B3 and B4 RNA in serum samples of 60 patients with T1DM and 120 healthy subjects; 25%, 23%, and 38% of patients with T1DM tested positive for Coxsackievirus B4 RNA, IgM against Coxsackievirus B4 and GAD65, and IgG against GAD65, respectively, compared with the 1%, 3% and 5% of the control group. Based on these results, the authors proposed that children in Iraq are at a risk of T1DM if they have been exposed to Coxsackievirus B4 [36]. 

### 2.3. Iran

Iran reported a relatively low prevalence of T1DM in 2019 [15]. In a case-control study, EVs in the serum of the newly diagnosed patients with T1DM were studied (Table 2). Interestingly, the EV infections were significantly higher in patients with T1DM than in otherwise healthy individuals. Serum samples were analyzed using RT-PCR and immunoassays to detect IgG and IgA. EV RNA was detected in 34% (12 out of 35) of T1DM patients, compared with 3% (1 out of 35) of controls. Moreover, 37% and 29% of T1DM patients tested positive for IgG and IgA against EVs, respectively, compared with 8% and 6% of controls. Thus, a significant correlation between T1DM and EVs was found in the studied population [37]. 

### 2.4. Sudan

Sudan is a large country in the MENA region with 12,000 children and adolescents with T1DM in 2019 [15]. A Sudanese study on T1DM conducted between 2005 and 2007 showed a significant increase in the total number of the affected children from 2800 to 3500. The increasing rate at which people were moving from rural to urban areas was thought to influence this rise in cases [38]. Most cases were diagnosed in winter. Moreover, EVs were investigated as possible causes of T1DM by analyzing the serological profile (IgG and IgM) of Coxsackieviruses using immunoassays; IgG and IgM were positive in 46% and 8%, respectively (Table 2). These notable seroprevalence profiles could explain the association between EV and T1DM [38]. 

### 2.5. Algeria, Morocco, and Tunisia

Algeria and Morocco, two large countries in the MENA region, have almost a similar T1DM prevalence. In 2019, approximately 33,000 and 30,000 children aged <20 years were diagnosed with T1DM in Algeria and Morocco, respectively [15]. Despite these high numbers, it is difficult to conclude whether T1DM was related to EV infections, owing to scarcity in literature. However, a recent cross-sectional study from Morocco reported an approximate 8% increase in the rate of hospitalization for T1DM patients between 2015 to 2018, and most cases were reported in spring, the season of EV infections in the MENA region. Unfortunately, no data on EVs and their relationship to T1DM in Morocco or Algeria are available [43]. However, in Tunisia, which shares its western border with Algeria, the role of EV in T1DM has been studied. In a case-control study, the presence of EV RNA was investigated in the plasma of 95 children and adults with T1DM and matched to 141 healthy subjects (Table 2). EV RNA was detected in a significantly higher number of patients with T1DM, compared to the control group (32% versus 8%). Moreover, EV RNA was more frequently detected in children than in adults (54% versus 15%, respectively). However, no association was noticed between EV RNA and GAD65 antibodies. These results suggested that EV infections are important environmental triggers of T1DM, and EV RNA is associated with T1DM in the Tunisian population, particularly in children [39]. 

### 2.6. Israel

Approximately 4000 children and adolescents were diagnosed with T1DM in Israel in 2019 [15]. The correlation between maternal EV antibodies and T1DM incidence was evaluated in Israel. Serum samples obtained from pregnant women from Israel, in addition to Estonia, Germany, Hungary, Lithuania, and Russia, as countries with a low incidence of T1DM, were analyzed for EV antibodies, using immunoassays and neutralization assays. Samples were also obtained from pregnant women from Finland and Sweden, as countries with a high incidence of T1DM. Israel reported the lowest incidence of T1DM and the highest levels of maternal EV antibodies, compared with all other countries, notably, Finland and Sweden. The frequency of EV infections in “the background population” may affect the susceptibility of children to the possible diabetogenic effects of EVs [44]. Furthermore, in countries with a high incidence of T1DM, maternal EV antibodies are decreasing and might have disappeared when children first contracted the infection [44]. Another recent study from Israel was conducted to determine if T1DM immunological markers and viral antibodies can be detected during winter in healthy pregnant non-diabetic women without a family history of diabetes; 107 maternal and cord blood sera from healthy pregnant non-diabetic women and their children at birth in two consecutive winter seasons were collected. These samples were examined for T1DM immunological markers and for antibodies against Coxsackievirus B3 and Rotavirus, using radioligand binding assays and immunoassays. A significant association was found between GAD65 autoantibodies and Rotavirus antibodies in both maternal and cord blood sera. Besides, Coxsackievirus B3, Rotavirus, and GAD65 antibodies were detected in the cord blood in 17, 22, and 5 pregnancies, respectively, but not in the maternal serum. The appearance of these antibodies in the cord blood but not in maternal samples was reported previously with viral and parasitic infections, and it is suggested to be an independent active humoral response to an insult of the fetal pancreas in the pre-diabetic stage [45,46,47]. In 1 of 20 pregnancies, Coxsackievirus B3 infection occurred after 20 weeks of gestation and was detected by an increase in the antibody titer against Coxsackievirus B3. Another pregnancy presented with similar findings for Rotavirus. These results indicate that T1DM immunological triggers occurred owing to viral infections during pregnancy [48].

## 3. Africa

Africa is one of the least affected regions of T1DM (Figure 1). Approximately 26,000 children and adolescents aged < 20 years were diagnosed with T1DM in 2019 [15]. Owing to this low prevalence, there is a dearth of information on T1DM, and its clinical and epidemiological features are poorly defined. In the last few years, different studies found that the incidence of T1DM was slowly increasing in Gabon or decreasing in Ghana. This low incidence of T1DM was owing to hygienic environmental practices, genetic variations, and high rates of helminthic infections [49,50,51]. 

### 3.1. Nigeria

The relationship between T1DM and EV was studied earlier in serum of 40 insulin-treated Nigerian diabetic patients, using complement fixation. A higher level of anti-Coxsackievirus A in 40 non-diabetic subjects was observed, compared with diabetic patients, with no significant difference in levels of antibodies directed toward Coxsackievirus B and other viruses (mumps and rubella). Thus, early exposure to these viruses may not be an important factor in T1DM etiology [52]. 

### 3.2. Congo

A recent study evaluated the activity of anti-Coxsackievirus B4 in the saliva of 181 patients with T1DM from Congo, Lebanon, and France. Those patients were matched to 120 healthy individuals. Using sero-neutralization assay, the saliva of T1DM Congolese patients, in addition to Lebanese and French T1DM patients, expressed a higher anti-Coxsackievirus B4 activity than that of individuals in the control group. Moreover, during the four years of this follow-up study, an increase in the activity of anti-Coxsackievirus B4 was reported in Congolese T1DM patients, compared with that in the control group. Furthermore, the titers of Coxsackievirus B4 neutralizing antibodies in saliva samples from Congolese patients were higher than those from Lebanese and French patients. These differences can be explained by environmental variations and early exposure to EVs in African countries [40].

### 3.3. Benin

In a case-control study, the neutralizing anti-Coxsackievirus B4 activity in the saliva and serum of 15 patients with T1DM was analyzed and matched to 8 healthy controls. The neutralizing activity was significantly higher in the saliva of patients with T1DM. However, no significant difference was found in the serum samples of both groups. Moreover, an imbalance in B cells and T helper cells was observed in T1DM patients but not in the control group. Taken together, these findings indicate that the activity of salivary anti-Coxsackievirus B4 in T1DM patients is associated with immune system dysregulation. Thus, the anti-Coxsackievirus B4 activity in the saliva can be used as a non-invasive marker to examine the viral pathogenesis of T1DM, especially in high-risk populations [41].

## 4. Asia

Asia is one of the highly affected regions with T1DM. Approximately 184,000 children and adolescents were living with T1DM in 2019, with 21,000 newly diagnosed cases each year [15]. 

### 4.1. India 

India has recorded the highest incidence (*n* = 16,000) and prevalence (*n* = 95,000) of T1DM in children aged <14 years worldwide [15]. However, the clinical and epidemiological features, including environmental triggers, are poorly characterized. EVs are widely reported in India as a major cause of CNS and gastrointestinal infections. Echoviruses and Coxsackievirus B are among the most common serotypes reported in CNS infections [53,54,55,56]. Despite these numbers, EV infections were not studied as a potential etiology of T1DM in India and further investigations are of utmost importance. 

### 4.2. China

China is one of the countries that recorded a high prevalence of T1DM in 2019 at 54,000 cases [15]. A study investigated the incidence trends of T1DM in Shanghai, East China, between 1997 and 2011 and found that the mean annual incidence rate of T1DM in children aged <14 years was 3.1 per 100,000 persons. A 14% increase in the mean annual incidence during the study period was reported. The possible role of EV infections, as causative agents of hand, foot, and mouth disease, which is common and reflects EVs’ circulation in Shanghai has also been discussed. They proposed that EVs could be potential triggers of T1DM in Shanghai, and this might explain the increased incidence of T1DM [57]. Another study investigating the role of infectious agents in the development of T1DM in China evaluated the relative risk of T1DM immediately after infections in 260 patients. Data were retrieved from the medical records system, and patients’ parents were interviewed to collect information on the incidence of infectious diseases 407 days before the onset of T1DM. During this period, 18% of patients had an infection. Respiratory infections such as the common cold were the most frequently reported infections (57%). Many were caused by viruses, notably EVs, which might have served as an etiological agent in the development of T1DM. However, further epidemiological and molecular studies are required to ascertain this relationship in China [58].

### 4.3. Japan

Approximately 4500 children and adolescents are living with T1DM in Japan [15]. Furthermore, fulminant T1DM, a different subtype of T1DM characterized by the rapid and complete destruction of pancreatic β-cells, accounts for approximately 20% of acute-onset T1DM and is reported mainly in adults [59]. T1DM in the Japanese population has been previously studied [60,61,62]. Moreover, the relationship between fulminant T1DM and EVs has also been investigated in Japan. In a case-control study, Imagawa et al. studied IgM, IgG, and IgA antibodies against EV, using ELISA, in 19 patients with recent-onset fulminant T1DM, 18 patients with recent-onset typical T1DM, and 19 healthy subjects (Table 2). Compared with patients with recent-onset typical T1DM and healthy subjects, IgA antibodies against EVs were significantly higher in patients with fulminant T1DM. Thus, patients with fulminant T1DM were more susceptible to EV infections and these infections might have had an etiological role in fulminant T1DM [63]. Moreover, Akatsuka et al. reported a case of a 39-year-old woman with fulminant T1DM. Among all investigated neutralizing antibodies against other viruses, only neutralizing antibodies for Coxsackievirus B4 had an 8-fold increase after 4 weeks of hospitalization. These results suggested that the onset of fulminant T1DM was associated with Coxsackievirus B4 infection [64]. Tanaka et al. studied whether EVs can induce accelerated β-cell dysfunction in patients with fulminant T1DM. Samples were taken from three patients who died from fulminant T1DM complications, within a few days after the onset of the disease. Using immunohistochemistry and RT-PCR, islet cells were examined for the presence of EVs. Interestingly, EV capsid protein was detected in all three samples with evidence of β-cell destruction. These findings suggested that EVs can result in the onset of fulminant T1DM [9].

### 4.4. Taiwan

Taiwan reported a low T1DM prevalence with approximately 3000 children and adolescents in 2019 [15]. EV infections were investigated as a possible cause of T1DM in Taiwan (Table 2). The medical records system had been reviewed for over 100,000 children and adolescents aged < 18 years with T1DM. Patients with T1DM diagnosed with EV infections between 2000 and 2008 were compared with those not infected with EVs. A significant association between the overall incidence of T1DM and EV infections was noted. Moreover, in the control group, the incidence of T1DM increased with age. The authors suggested that a preventive vaccine against EVs could reduce the incidence of T1DM. In this study, the diagnosis of EV infection depended on the medical records, and evidential EVs serotypes were not specified. Some EV infections were diagnosed based solely on the clinical manifestations, such as herpangina and hand, foot, and mouth disease, and not on molecular or serological test results. However, with such population numbers in the study (>100,000 for both arms), the relationship between T1DM incidence and EVs cannot be denied in the Taiwanese population [42]. 

### 4.5. Russia

Russia is one of the countries with a relatively lower incidence of T1DM than some European or Asian countries [15]. Viskari et al. analyzed the relationship between the frequency of EV infections and T1DM incidence in seven countries, using immunoassays and neutralization assays. Two of these countries had a high incidence of T1DM (Finland and Sweden) and the remaining five had a low or intermediate incidence of T1DM (Estonia, Germany, Hungary, Lithuania, and Russia). Interestingly, countries with a low T1DM incidence, like Russia, reported significantly more frequent EV antibodies than countries with a high T1DM incidence [65].

## 5. Cuba

Cuba is one of the countries that reported a low incidence of T1DM and high circulation of EVs. A case-control study analyzed neutralizing antibodies to Coxsackievirus B and Echovirus in the serum of three groups: 33 newly diagnosed patients with T1DM, 43 healthy children with a family history of T1DM without islet cell antibodies (ICA), and 57 controls (Table 2). Titers of neutralizing antibodies to Echovirus 4 were significantly higher in the diabetic group than in the control group without significant differences between the diabetic children and healthy children of diabetic parents. These findings suggested that there was a significant relationship between T1DM and the presence of neutralizing antibodies to Echovirus 4, indicating that EV could be an etiological agent for T1DM [66]. Echovirus 16 was also found to be associated with T1DM autoantibodies in another Cuban study (Table 2) [67]. In this case-control study, serum from 38 children and adults with T1DM were matched to serum from 80 control subjects. An important association was noted between neutralizing antibodies to Echovirus 16 and T1DM immunological markers, GAD antibodies, ICA, and insulin autoantibodies (IAA). Compared with 0% in healthy subjects, ICA, IAA, and GAD65 seroconversion was positive in 92%, 45%, and 29% of Echovirus 16-infected subjects in the convalescent stage, respectively. Thus, the possible effects of Echovirus 16 on the process of autoimmune β-cell disruption could make EV infections an important risk factor for T1DM [67]. In another case report of a 12-year-old girl with clinical manifestations in favor of viral meningitis, Echovirus 30 was found to be the causative agent, using immunoassay techniques. However, the fasting blood glucose tested at the time was within normal range. Two months later, she presented with the signs and symptoms of T1DM and positive for ICA. As the seropositivity of ICA occurred a few months after the EV infection, Echovirus 30 was suggested of playing an important role in T1DM etiology [68]. Another Cuban study (Table 2), determining whether the presence of EV RNA was associated with T1DM and insulin-producing cells’ autoantibodies, found that the detection of EV RNA is significantly associated with newly diagnosed patients with T1DM and positive ICA results. Moreover, newly diagnosed T1DM children with positive EV RNA results appeared to have more complications, like diabetic ketoacidosis, and higher ICA levels. Thus, this relationship between T1DM and EVs found that the Cuban population is similar to that in the European population, despite differences in the rate of EV circulation and the incidence of T1DM between the two populations [69]. 

## 6. Conclusions and Future Directions

Although the incidence of T1DM is rising globally, intercontinental variation exists, with some regions having a higher incidence than others. Recently, many developing countries have been experiencing a rapid increase in the incidence rate with unexplained reasons yet to be thoroughly investigated. To face these rising T1DM trends in the concerned countries, focused epidemiological studies are warranted. Environmental triggers are also essential while studying EVs and their potential link to the onset and pathogenesis of T1DM. Future studies on T1DM, and its potential causal relationship with EVs, can lead to a better comprehension of the disease and eventually can aid in its prevention. Focused molecular and epidemiological studies on T1DM in some regions that recorded a high incidence and prevalence of T1DM, such as in the MENA regions and India, and the possible involvement of enteroviral infections that may increase T1DM incidence in those regions, are needed to bridge the gap of knowledge on the relationship between EVs and T1DM.

## Figures and Tables

**Table 1:** Top 10 countries with the highest incidence rates of type 1 diabetes [15].

Rank	Country	Incidence Rate of T1DM for Patients Aged 0–14 Years (Per 100,000 Per Year)
1	Finland	62.3
2	Sweden	43.2
3	Kuwait	41.7
4	Norway	33.6
5	Saudi Arabia	31.4
6	Canada	29.9
7	United Kingdom	29.4
8	Qatar	28.4
9	Ireland	27.5
10	Denmark	27

**Table 2:** Summary of studies that found a relationship between enterovirus infections and type 1 diabetes.

Study	Year of Publication	Type of Study	Population	Country	Method of Detection	Serotype Detected
Bilal et al. [36]	2019	Case-control	Aged < 17 years (60 T1DM cases and 120 controls)	Iraq	- ELISA (IgG and IgM) - RT-PCR	CVB 3 CVB 4
Samani et al. [37]	2017	Case-control	Aged < 30 years (35 T1DM cases and 35 controls)	Iran	- ELISA (IgG and IgM) - RT-PCR	-
Emad et al. [38]	2011	Questionnaire-based	101 children (age not specified)	Sudan	- ELISA (IgG and IgM)	-
Boussaid et al. [39]	2017	Case-control	- Children (2–15 years) and adults (16–48 years) - 95 T1DM cases and 141 controls	Tunisia	RT-PCR	-
Badia-Boungou et al. [40]	2017	Case-control	Mean age, 14 years (181 T1DM cases and 135 controls)	Congo Lebanon France	Neutralization assay	CVB
Nekoua et al. [41]	2018	Case-control	Mean age, 23 years (15 T1DM cases and 8 controls)	Benin	Neutralization assay	CVB 4
Lin et al. [42]	2015	Retrospective	Aged < 18 years	Taiwan	NA	-
Imagawa et al. (64)	2005	Case-control	19 patients with fulminant T1DM, 18 patients with typical T1DM, and 19 healthy control	Japan	ELISA (IgM, IgA, and IgG)	-
Diaz-Horta et al. (67)	2001	Case-control	33 T1DM patients at diagnosis, 43 healthy children of parents with T1DM without ICA, and 57 healthy subjects	Cuba	Neutralization assay	Echovirus 4
Cabrera-Rode et al. (68)	2003	Case-control	118 children and adolescents (38 T1DM cases and 80 controls)	Cuba	Neutralization assay	Echovirus 16
Sarmiento et al. (70)	2007	Case-control	Aged 1–15 years (34 newly diagnosed T1DM, 31 positive ICA, and 32 negative ICA cases and 194 controls)	Cuba	RT-PCR	-

T1DM: Type 1 diabetes mellitus. ICA: Islet cell antibodies. ELISA: Enzyme-linked immunosorbent assay. RT-PCR: Real-time polymerase chain reaction. CVB: Coxsackievirus B. NA: Not Available.
